# Spatio-Temporal Sequential Memory Model With Mini-Column Neural Network

**DOI:** 10.3389/fnins.2021.650430

**Published:** 2021-05-28

**Authors:** Yawen Lan, Xiaobin Wang, Yuchen Wang

**Affiliations:** ^1^School of Computer Science and Engineering, University of Electronic Science and Technology of China, Chengdu, China; ^2^School of Information Engineering, Southwest University of Science and Technology, Mianyang, China

**Keywords:** memory model, mini-column structure, excitatory neurons, inhibitory neurons, spatio-temporal sequence, spike-based encoding

## Abstract

Memory is an intricate process involving various faculties of the brain and is a central component in human cognition. However, the exact mechanism that brings about memory in our brain remains elusive and the performance of the existing memory models is not satisfactory. To overcome these problems, this paper puts forward a brain-inspired spatio-temporal sequential memory model based on spiking neural networks (SNNs). Inspired by the structure of the neocortex, the proposed model is structured by many mini-columns composed of biological spiking neurons. Each mini-column represents one memory item, and the firing of different spiking neurons in the mini-column depends on the context of the previous inputs. The Spike-Timing-Dependant Plasticity (STDP) is used to update the connections between excitatory neurons and formulates association between two memory items. In addition, the inhibitory neurons are employed to prevent incorrect prediction, which contributes to improving the retrieval accuracy. Experimental results demonstrate that the proposed model can effectively store a huge number of data and accurately retrieve them when sufficient context is provided. This work not only provides a new memory model but also suggests how memory could be formulated with excitatory/inhibitory neurons, spike-based encoding, and mini-column structure.

## 1. Introduction

Memory plays a critical role in human cognition, and emerging experimental results show that the formulation of memory is extremely complex involving multiple brain regions. There are many different memory types and can be classified as declarative memory and non-declarative memory (Glenberg, 1997; Squire, 2004). Both types of memory require the storage and retrieval of sequence information. It is evident that the ability to memorize and predict sequential information is critical to several cognitive tasks, such as speech recognition, natural language processing, motor control, and etc. (Cui et al., 2016; Lee et al., 2019; Lee and Li, 2020). However, the exact mechanism of how the brain formulates sequential memory remains an open question. Over the past decades, researchers from neuroscience and machine learning have devoted significant effort to explore the underlying mechanisms of sequential memory, and proposed many related models from the perspective of machine learning (Eichenbaum, 2017; Kitamura et al., 2017; Rolls and Mills, 2019; Herweg et al., 2020; Josselyn and Tonegawa, 2020).

The most popular machine learning method for sequential information processing is the recurrent neural networks (RNNs). Characterized by feedback links and internal state (Memory), RNNs have been successfully implemented in many sequence applications (Zhang et al., 2020a,b). However, traditional RNN suffers from the long-term dependence problem in which the backpropagation gradient will vanish after a long sequential span (Hochreiter and Schmidhuber, 1997). To resolve this problem, long short-term memory (LSTM) (Hochreiter and Schmidhuber, 1997) introduces a memory cell to RNN for efficient storage of sequences with bigger and varying time-scales. The time delay neural networks (TDNNs) (Lang et al., 1990) are another way for sequence information processing, which organize sequential memory information in a multilayer feedforward structure. The current machine learning methods obtain impressive performance in sequence information processing and prediction. However, they separate the training and testing data sets and make an assumption that the training and testing data sets have similar statistic features. This is an unrealistic assumption of the world whereby the unseen data is noisy and changes dynamically (Cui et al., 2016). In addition, tuning the parameters of the current machine learning methods is a very difficult task, and this process is timing-consuming. These limitations promoted us to develop brain-inspired memory models.

Although the exact mechanism of sequence memory formation in the brain remains an open question, the biologically plausible spiking neuron models (Maass, 1997; Gerstner, 1998; Izhikevich, 2003), spike-based encoding, and learning algorithms (Bi and Poo, 1998; Gütig and Sompolinsky, 2006; Zhang et al., 2018; Pan et al., 2019, 2020a; Pokorny et al., 2020) are relatively well-studied and understood. Traditionally, the firing rate of neurons is assumed to encode the information. However, these rate-based encoding methods cannot explain the rapid process in the pathways of visual (Meister et al., 1995; Neuenschwander and Singer, 1996), auditory (Decharms and Merzenich, 1996), and olfactory (Wehr and Laurent, 1996). Increasing evidence supports the spike-based temporal coding that information is represented by the firing timing of spikes.

Spiking neurons are proposed to emulate the mechanism of how biological neurons deal with the spatio-temporal spike information (Gerstner, 1998). Spiking neurons can be used to construct spiking neural networks (SNNs). The synaptic weights in SNNs will change in the acquisition of new knowledge. Various spike-based methods have been proposed to update the synaptic weights in SNNs. They can be divided into supervised and unsupervised algorithms. The tempotron (Gütig and Sompolinsky, 2006) is one of the most popular supervised learning algorithms in SNNs, and has been widely used (Wu J. et al., 2018). It trains the synaptic weights to make the spiking neuron output a spike in response to the correct input, and otherwise keeps silent. One drawback of tempotron is that only one output spike can be controlled. To resolve this problem, many learning algorithms have been proposed to train the spiking neurons to output multiple spikes, such as remote supervised method (ReSuMe) (Ponulak and Kasiński, 2010) and membrane potential driven aggregate label learning algorithm (MPD-AL) (Zhang et al., 2019). Through updating the synaptic weights, ReSuMe can train a spiking neuron to output precisely timed spikes, and MPD-AL can train a spiking neuron to emit a desired number of spikes. Recently, there are many supervised learning algorithms have been proposed for deep SNNs, and achieve good performance on complex and large data set (Lee et al., 2016; Wu Y. et al., 2018, 2019, 2020; Pan et al., 2020b; Panda et al., 2020; Wu J. et al., 2020). In the area of unsupervised learning rules, the spike-timing-dependent plasticity (STDP) (Bi and Poo, 1998) is one of the most popular rules. According to STDP, synaptic plasticity depends on the firing times between the pre- and postsynaptic neurons. STDP is proved to be able to train distinct patterns in an unsupervised manner and has been widely used in many real-world applications (Masquelier and Thorpe, 2007; Masquelier et al., 2009; Wu J. et al., 2019).

Due to the significant progress in encoding and learning of SNNs, it is possible to leverage the advantage of SNN to build an SNN-based memory model. Horzyk uses associative pulsing neurons (APNs), a simplified spiking neuron model, to construct a spatio-temporal sequential memory model called active neuro-associative knowledge graphs (ANAKG) (Horzyk, 2014; Horzyk and Starzyk, 2017). Experimental results demonstrate that ANAKG can effectively store and retrieve sequential data, such as sentences. To further improve the memory capacity and retrieval performance, improvements have been proposed with mini-column structure (Starzyk and Horzyk, 2019a) and synaptic delay plasticity (Starzyk et al., 2019b). The spatio-temporal memory (STM) (Hu et al., 2016) model employs spiking neurons and neuroscience findings to explore how the brain formulates memory with STDP and hierarchical structure. Researchers have implemented the STM model on hardware to test its performance (Liu et al., 2019). He et al. (2019) also construct an associative memory system through SNNs, in which the formulation of memory consists of structure formation and parameter training. In addition, inspired by the famous hierarchical temporal memory (HTM) model (Cui et al., 2016), Liang et al. (2020) propose a temporal-sequence memory model with mini-column structure, and music memory and retrieval are selected as a real-world application to verify the performance. The threshold phasor associative memory (TPAM) network is another memory model (Frady and Sommer, 2019), which is inspired by the traditional Hopfield networks. The TPAM networks can be further transformed to SNN through a “phase-to-timing” mapping.

Although various memory models have been proposed with biologically plausible spiking neurons, the memory capacity can be further improved. Take one of the best performing model for instance, in the experiments of the STM model (Hu et al., 2016), only one word can be remembered and retrieved after hundreds of training iteration. In this work, we still leverage the advantage of spiking neurons in processing spatio-temporal data and propose a new memory model based on the structure of the neocortex. The proposed model is structured by different mini-columns which are used to encode and represent different memory items. Each mini-column consists of many spiking neurons, and the fire of different neurons in one mini-column depends on the context of previous inputs. During the learning process, the STDP rule is applied to train the memory model in a one-shot learning manner. Furthermore, to improve the retrieval accuracy, the global-based inhibitory neuron is also employed to prevent incorrect retrievals. Extensive experiments have been conducted to evaluate the performance of the proposed model, and the results show that the proposed model can effectively store a huge number of data and retrieve them with high accuracy. This work not only provides a new memory model but also suggests of how the brain formulates memory with excitatory/inhibitory neurons, spike-based encoding, and mini-column structure.

## 2. Methods

In this section, we firstly introduce the employed spiking neuron model. Then, the proposed memory model and algorithms are described in detail.

### 2.1. Spiking Neuron Model

Many mathematical spiking neuron models have been proposed to emulate the dynamics of biological neurons, such as Hodgkin–Huxley model (HH), integrate-and-fire (IF), spike response model (SRM), and etc. (Maass, 1997; Gerstner, 1998; Izhikevich, 2003). Among these models, the current-based leaky integrate-and-fire model is biologically plausible and mathematically tractable. Hence, it is employed in our memory model.

The membrane voltage of a spiking neuron is represented by *V*(*t*), which is initialized as resting potential *V*_*rest*_(*t*) = 0. The spikes generated by the presynaptic neurons will cause a Postsynaptic potential (PSP) in the postsynaptic spiking neuron. The postsynaptic neuron integrates the input spikes over time, and output a spike when the cumulative PSPs reach the firing threshold ϑ. After that, the postsynaptic neuron enters a period called the refractory period, in which the spiking neuron is much harder to fire a spike. The dynamics of the neuron can be express as Equation (1).

(1) $$\begin{array}{l} V\left(t\right)=V_{rest}+\overset{N}{\underset{i=1}{\sum }}\omega_{i}\underset{t_{i}^{j}<t}{\sum }K\left(t-t_{i}^{j}\right)-\underset{t_{s}^{j}<t}{\sum }\eta \left(t-t_{s}^{j}\right), \end{array}$$

where $t_{i}^{j}$ is the firing time of *j*th spike from the presynaptic neuron *i*, and ω_*i*_ denotes the synaptic weight from presynaptic neuron *i* to postsynaptic neuron. $K\left(t-t_{i}^{j}\right)$ is the kernel of the PSP function defined as Equation (2).

(2) $$\begin{array}{l} K\left(t-t_{i}^{j}\right)\;=\;V_{0}\left[\exp\left(-\frac{t-t_{i}^{j}}{\tau_{m}}\right)-\exp\left(-\frac{t-t_{i}^{j}}{\tau_{s}}\right)\right],t-t_{i}^{j}>0, \end{array}$$

**Figure 1:**
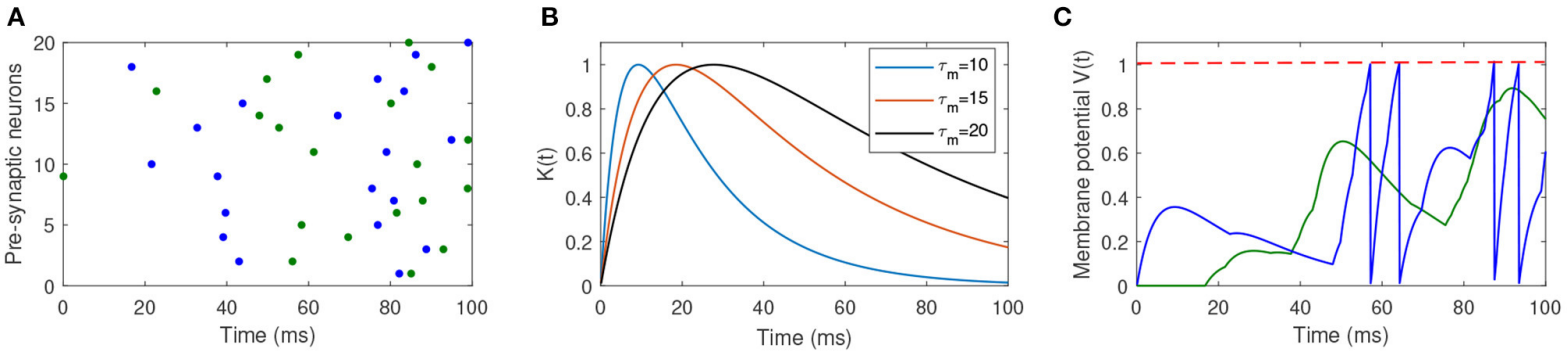
Illustration of the dynamics of the used spiking neuron. **(A)** Two different input spike patterns, which are distinguished by different colors. **(B)** The shape of the PSP with different values of τ_*m*_ and τ_*s*_. **(C)** The membrane potential of the postsynaptic neuron in response to different input spike patterns. The blue input spike pattern makes the postsynaptic neuron fire four spikes, while the green input spike cannot make the neuron fire a spike.

The shape of PSP is governed by the parameters of *V*_0_, τ_*m*_, and τ_*s*_. *V*_0_ is used to normalized the maximum value of PSP to 1, τ_*m*_ and τ_*s*_ are the membrane and synaptic time constants, respectively. The last kernel of Equation (1) describes the refractory process, which is further detailed as

(3) $$\begin{array}{l} \eta \left(t-t_{s}^{j}\right)=\vartheta \cdot \exp\left(-\frac{t-t_{s}^{j}}{\tau_{m}}\right),\;t-t_{s}^{j}>0, \end{array}$$

where ϑ is the firing threshold, and $t_{s}^{j}$ is the firing time of *j*th spike generated from the postsynaptic neuron. Figure 1 shows the dynamics of the spiking neuron.

### 2.2. Network Architecture

**Figure 2:**
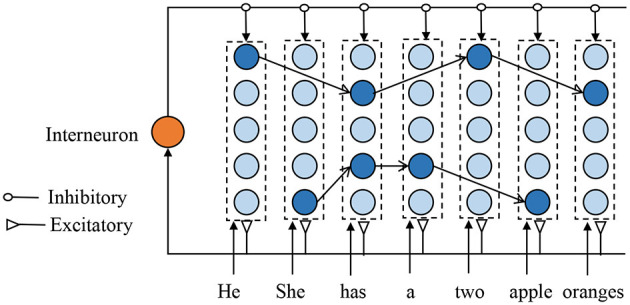
Illustration of the proposed memory model structured with many mini-columns. Each column, comprises of spiking neurons, represent one memory item, and the firing neuron in each column depend on the previous input context. For clarity, the weak connections are not shown in this figure.

Figure 2 depicts the proposed SNNs-based episodic memory model, which is inspired by the columnar organization of the human neocortex (Mountcastle, 1997) and HTM model (Cui et al., 2016). Each column consists of several biological spiking neurons and represents a single memory item (such as one letter or one word). Although the neurons in one column are duplicates of each other and encode for the same information, their synaptic connections are very different to represent the different contexts. The firing neuron in each column is decided by the previous input context. Assume two sentences: *A-C-E-G* and *B-C-D-F*, there are two different neurons that present different “*C*” in different sentences. Each neuron in the proposed model has three inputs: excitatory inputs from the feedforward sensory data, excitatory inputs from the laterally connected neurons, and inhibitory inputs from the interneurons.

The feedforward signal is used to activate the corresponding spiking neuron, and this is very important to perform STDP learning and make a prediction. Whenever there is an input signal, the global-based interneuron generates a spike and provides an inhibitory signal to prevent neurons from making the wrong predictions. Due to the excitatory lateral inputs, the neuron can fire a spike even without feedforward inputs, which contributes to sequence retrieval and prediction. In addition, when there is a feedforward spike, the neuron with stronger lateral input will generate an earlier spike and prevents other neurons from firing.

### 2.3. Synaptic Plasticity Rule

**Figure 3:**
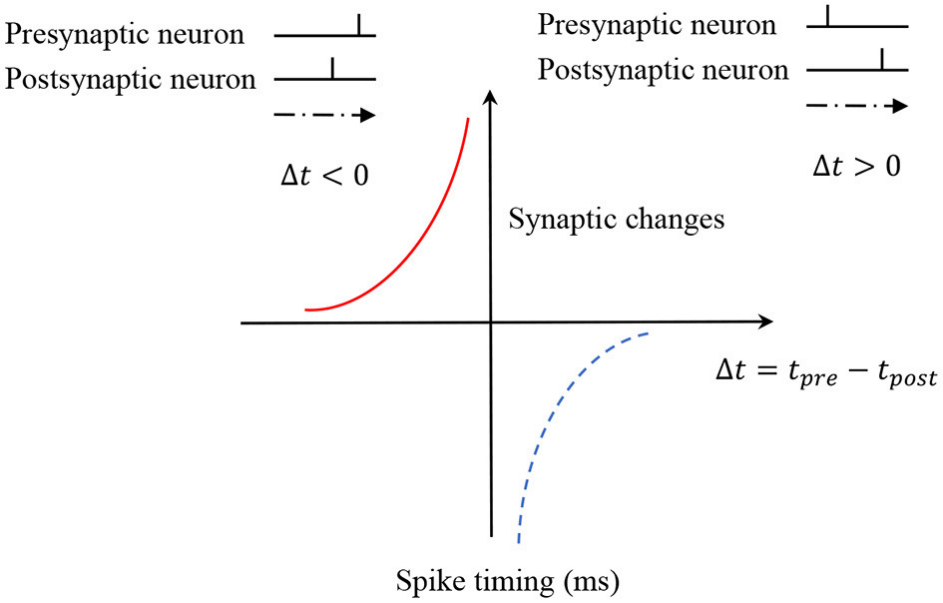
The STDP learning rule. STDP consists of two update processes: LTD and LTP.

Synaptic plasticity is crucial in knowledge acquirement and memory formation. There are various spike-based learning algorithms in SNNs, and the STDP learning rule is selected to train the SNNs-based model. According to the STDP learning rule, if the presynaptic neuron fires a spike earlier than the postsynaptic neuron, a long-term potentiation (LTP) will be induced in the synapse. On the other hand, an inverse spike order between the presynaptic neuron and postsynaptic neuron leads to long-term depression (LTD) of the synapse. Therefore, the modification of the synapse can be defined as a function of the firing times of presynaptic and postsynaptic neurons, and typically the STDP function is defined as

(4) $$\begin{array}{l} \Delta \omega_{i}=\;\{\begin{array}{l} A^{+}\cdot \text{exp}(\frac{-s}{\tau^{+}})\text{ if }s>0\\ A^{-}\cdot \text{exp}(\frac{-s}{\tau^{-}})\text{ if }s<0, \end{array} \end{array}$$

where ω_*i*_ the synaptic weights from presynaptic neuron *i* to the postsynaptic neuron, *A*^+^ and *A*^−^ are the parameters that control the amplitudes of synaptic changes. *s* = *t*_*j*_ − *t*_*i*_ denotes the difference of firing times between two neurons. Figure 3 is used to illustrate the learning mechanism of STDP. In our model, we only consider memory formation and neglect the forgetting process. Therefore, only the LTP updates of STDP are used in this work.

According to the mechanism of the STDP learning rule, the synaptic weights between two neurons are decided by the firing time interval between the presynaptic and postsynaptic neurons. In this work, the synaptic connections which are formed by the adjacent firing neurons are defined as *strong connections*, while the others form *weak connections*.

### 2.4. Sequence Storage

Given a sequence data set 𝕊 = {*S*^1^, *S*^2^, …, *S*^*N*^}, in which one sequence can be expressed as $S^{n}=\left\{E_{1}^{n},E_{2}^{n},\ldots ,E_{k}^{n}\right\}$. $E_{i}^{n}$ is one of the memory items in sequence *S*^*n*^, and *k* is the length of the sequence. In order to explain how the proposed model store (memory) sequence information, we first summarize the main steps and then explain the detailed process using an example.

**Step 1**. Continuously read the memory items of the input sequence, and add a new mini-column if the item is not represented by the existing mini-columns.
**Step 2**. Reuse the overlapping episodes that have previously been stored, and establish a new connection for the new memory sequences. In the following, the reused overlapping episodes are defined as ROE (Reused Overlapping Episode).
**Step 3**. Update the weights of new synapses between all the predecessor-successor neurons using the STDP rule.

Assuming that the memory model has stored the following sentences. *Mike didn't really know this. Mike really knows how to cook fish. Don't cook these wild greens*. The new input sentence is *Mike didn't really know how to cook these wild greens in spicy*. In the existing model, there are no corresponding columns to items “*in*” and “*spicy*.” According to Step 1, the model first adds two mini-columns to encode memory items of “*in*" and “*spicy*.”

**Figure 4:**
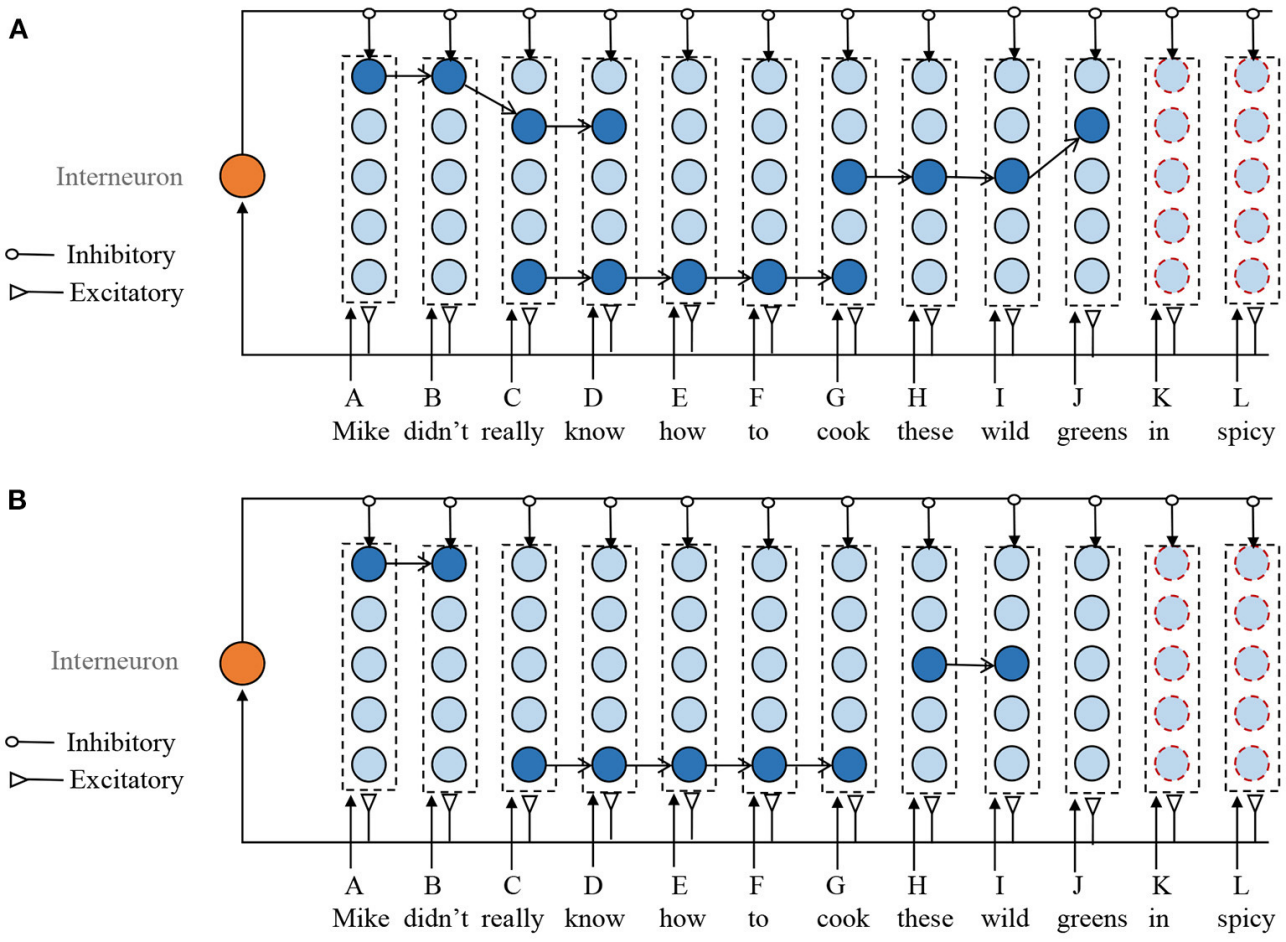
Illustration of the sequence storage process. It mainly consists of three steps. (1) Add new mini-column to represent the unseen memory items. (2) Obtain several ROEs without overlapping. (3) Update the synaptic efficiency according to the STDP learning rule. **(A)** Overlapped ROEs. **(B)** ROEs without overlap. For clarity, the weak connections are not shown in this figure.

Next is to find the overlapping episodes that have previously been stored. The selection of ROE should follow the following principles. First, the synapses between two adjacent neurons in the selected sub-sequence should have a strong connection. Second, if the neuron in a selected ROE is the end of any previously stored sequences and it is not yet the end of the new input sequence, then this neuron should be removed from the ROE. For example, as shown in Figure 4A, in the ROE of *G-H-I-J, J* is the end of the previously episodic (*Don't cook these wild greens*.). However, it is not the end of the new input sequences. Therefore, J should be deleted from the ROE of *G-H-I-J*. At last, when the selected ROEs overlap with each other, the overlapping neurons from the shorter ROE are deleted. For example, ROEs of *A-B-C-D* and *C-D-E-F-G* have the same sub-sequence of *C-D*, then the ROE of *A-B-C-D* is reduced to *A-B*. Although removing the overlapped mini-columns from larger ROE is also possible, it leads to fragmentation of the stored episodes.

Through the above steps, as shown in Figure 4B, we obtain several ROEs without overlapping mini-columns. Next, we should connect these ROEs together with other selected mini-columns that do not involve in any ROEs to form a connection for the new input sequence. The neuron in the mini-column is selected to make a combination with ROEs, whose number of input and outgoing connections are the smallest. Then, the STDP learning rule is applied to the newly added connections.

### 2.5. Sequence Retrieval

In this part, we use an example to introduce how the proposed memory model performs sequential retrieval with part of the contextual information. Assuming the memory model has stored two sentences: *A-B-C-D* and *A-B-E-F*. When the context *A-B-C* is presented, the memory model is expected to successfully recall *A-B-C-D* while does not retrieve the sequence *A-B-E-F*.

**Figure 5:**
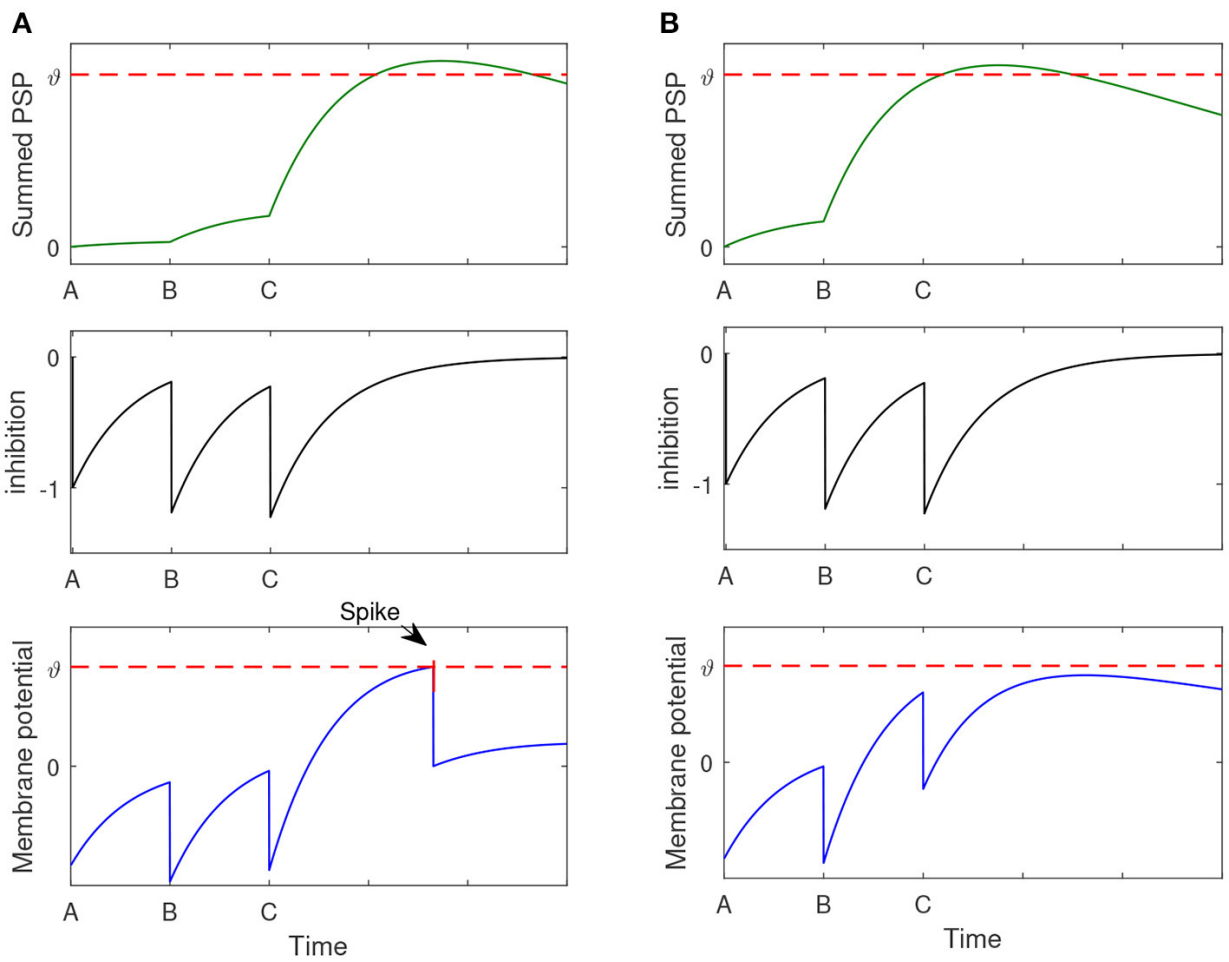
The process of sequence retrieval. The memory model has stored two sentences: *A-B-C-D* and *A-B-E-F*. When the context *A-B-C* is presented, the memory model can successfully recall *A-B-C-D* and it will not retrieve the sequence *A-B-E-F*. **(A)** The membrane potential dynamics of neuron “*D.”* **(B)** The membrane potential dynamics of neuron “*E”*.

Let's first analyze how can the proposed model successfully recall *A-B-C-D* with the context input *A-B-C*. Due to the storage process, there are excitatory connections between memory items of *A, B, C*, and *D*. When *A, B, C* input to the memory model one by one, the corresponding neurons fire spikes and transmit them to the ‘*D'* neuron. As shown in the top panel of Figure 5A, neuron “*D”* cumulates the effect of input spikes. The middle panel of Figure 5A shows the inhibitory signal produced by the interneuron. The down panel shows the membrane potential of the neuron “*D,”* which integrates the signals from both the excitatory and inhibitory inputs. We can see that the Neuron “D” fires a spike, and this means the element “*D”* can be successfully retrieved.

On the other hand, how to avoid the recall of *A-B-E-F* relies on another function of the memory model. In the storage process, there are excitatory connections between *A, B*, and *E*. As shown in the top panel of Figure 5B, the input of A, B may induce a heavily cumulated PSP in neuron “*E”* so that it can fire a wrong spike. However, this problem can be avoided by the utilize of the global inhibitory interneurons. When “*C”* inputs, the global inhibitory neuron will be activated, and send an inhibitory signal to prevent the firing of neuron “*E.”* The down panel of Figure 5B shows the membrane potential dynamics of neuron “*E,”* which does not generate the wrong spike.

## 3. Experiments

In this section, extensive experiments are conducted to verify the performance of the proposed memory model. Firstly, the employed data sets will be introduced. Then, the evaluation measure of memory performance is described. Finally, we report and analyze the experimental results.

### 3.1. Datasets

To verify the performance of the proposed memory model, we first conduct experiments on a small data set that consists of nine sentences. Table 1 shows the nine sentences (Starzyk et al., 2019b). This data set is selected as an example to demonstrate the memory capability and make a comparison with other related models.

**Table 1:** Example of sentences.

**No**.	**Content of sentence**
1	I have a monkey.
2	My monkey is lovely.
3	My monkey is very small.
4	It likes to sit on my head.
5	It is very lovely.
6	It is also very clever.
7	It can jump very quickly.
8	It learns very quickly.
9	I also have a small dog.

To evaluate the capability of our model on large data sets, the Children's Book Test (CBT) is selected. The CBT dataset contains about 9,000 different words and 19,000 sentences with at least 10 words. We use this dataset to test the proposed model on involved parameters.

### 3.2. Retrieval Quality Evaluation

There are many evaluation measures for memory retrieval or related tasks, and we apply the Levenshtein distance as it has been widely used in the research area of memory capability. In this work, the Levenshtein distance measures the required minimum number of word operations (including insertions, deletions, or substitutions) so that the recalled sentence can be transformed into the training sentence.

The Levenshtein distance between string *a* (of length |*a*|) and string *b* (of length |*b*|) is defined as *lev*_*ab*_(|*a*|, |*b*|) where:

(5) $$\begin{array}{l} {\text{lev}}_{a,b}\left(i,j\right)=\{\begin{array}{ll} \text{max}\left(i,j\right) & \text{if min}\left(i,j\right)=0,\\ \text{min}\{\begin{array}{l} {\text{lev}}_{a,b}\left(i-1,j\right)+1\\ {\text{lev}}_{a,b}\left(i,j-1\right)+1\\ {\text{lev}}_{a,b}\left(i-1,j-1\right)+1_{\left(a_{i}\neq b_{j}\right)} \end{array} & \text{ otherwise} \end{array} \end{array}$$

Here, *lev*_*a, b*_(*i, j*) denotes the distance between the first *i* words of string *a* and the first *j* words of string *b*. The 1_(_*a*__*i*_ ≠ *b*_*j*_)_ is a indicator function that equals to 0 when *a*_*i*_ ≠ *b*_*j*_, and equals to 1 otherwise. According to the definition of the Levenshtein distance, a smaller Levenshtein distance indicates a higher similarity between string *a* and string *b*. Therefore, the quality of memory retrieval can be evaluated by the Levenshtein distance.

### 3.3. Memory Example and Analysis

The first experiments are conducted on the small data set as shown in Table 1. In the learning phase, the nine sentences are presented to the memory model and trained by the learning method described in 2. After that, we test the performance of the memory capability by randomly presenting part of the sentence to see whether the whole sentence can be retrieved. Table 2 shows the retrieval results of our method and the existing two typical methods: SDAKG and ANAKG.

**Table 2:** Memory retrieval performance of different models.

**Inputs**	**Response of SDAKG**	**Response of ANAKG**	**Response of our model**
I	I have a monkey I have also a small dog	I have also a monkey small dog	I have a monkey I also have a small dog
My	My monkey is very small My monkey is lovely	My	My monkey is very small My monkey is lovely
It	It is very lovely It is also very clear It likes to sit on my head It can jump very quicky It learns quickly	It	It is very lovely It is also very clear It likes to sit on my head It can jump very quicky It learns quickly
I have	I have a monkey I have also a small dog	I have also a monkey small dog	I have a monkey
I have a	I have a monkey	I have also a monkey small dog	I have a monkey
It is	It is very lovely It is also very clear	It is	It is very lovely It is also very clear
It can	It can jump very quickly	It can jump very quickly	It can jump very quickly

From Table 2, we can find that all the three models work well with inputs (part of the learning sentence). For example, with the input “It can,” all models successfully retrieved the whole sentence “It can jump very quickly.” However, in a complex situation, the retrieval performance of the proposed model outperforms the ANAKG and SDAKG models. For example, when “I have” is presented to the memory model, the response of SDAKG and ANAKG are all wrong, while the proposed model can retrieve the whole sentence correctly. In this experiment, the better performance of our model is due to the role of the global inhibitory neuron. Every input feedforward signal will activate the interneuron to provide an inhibitory signal so that the wrong prediction can be mediated. For example, with the input of the word “have,” the neuron represents “also” will be inhibited. However, the other two models don't have a similar mechanism.

### 3.4. Memory Performance on Large Dataset

In this part, extensive experiments are conducted on the CBT data set to thoroughly verify the capability of the proposed memory model. We first compare our model against competitive methods, namely, LSTM and ANAKG. Then we investigate the effect of different parameters on memory retrieval performance.

#### 3.4.1. Comparison With Other Works

**Figure 6:**
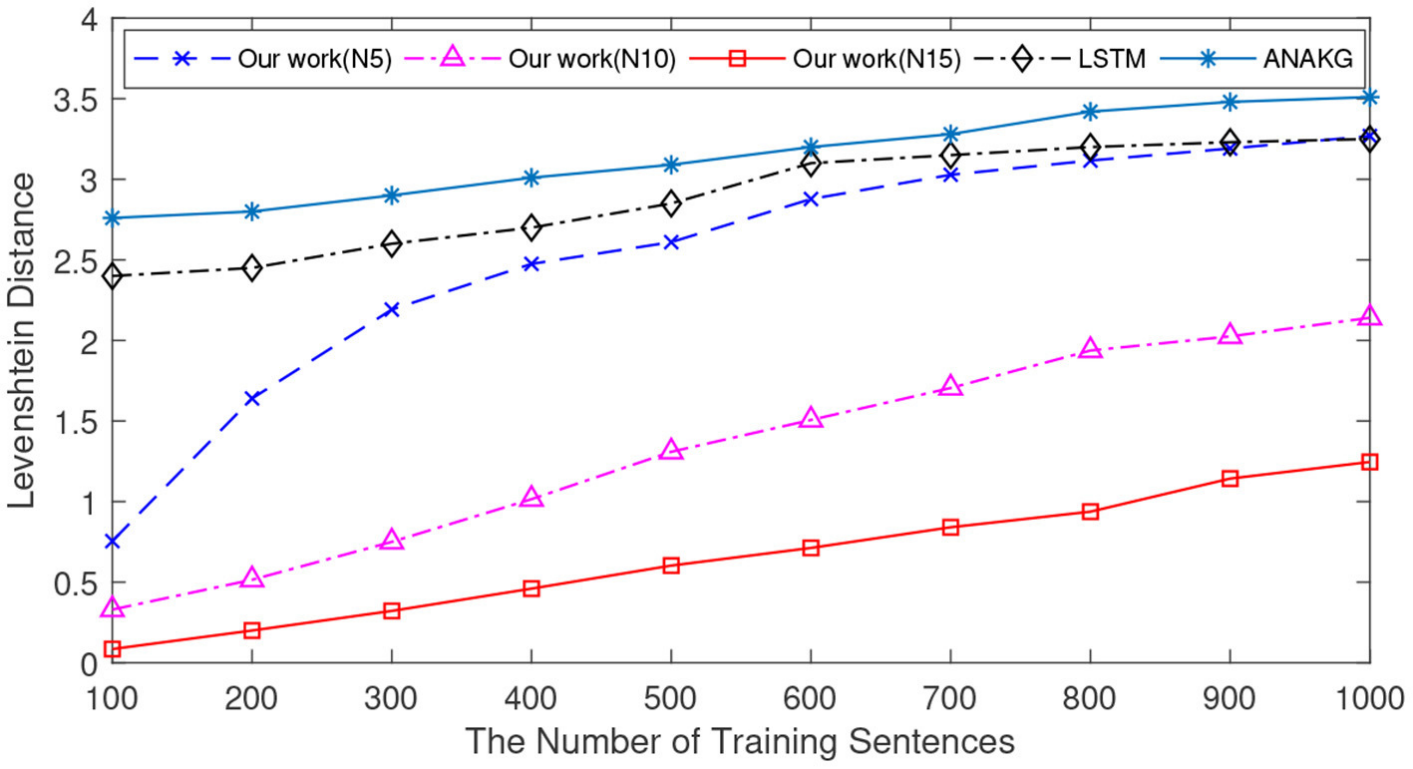
The memory retrieval performance of the LSTM, the ANAKG, and our proposed model with different number of sentences. Our model is trained with different column size and “N5” means there are 5 neurons in each column.

In these simulations, the models are trained to remember a different number of sentences from the CBT dataset. The number of the sentences various from 100 to 1,000 with an interval of 100. During the training process, we present the first 10 words of each sentence to different models. After learning, the first six words from each sentence are used as input to verify the retrieval performance. For each number of sentences, 10 independent experiments are conducted. The mean Levenshtein distances of different models are calculated and reported in Figure 6.

Figure 6 shows the memory retrieval performance of the LSTM, the ANAKG, and our model with a different number of neurons (5, 10, and 15) in each mini-column. The retrieval performance is indexed by the Levenshtein distance. The performances of ANAKG and LSTM have been reported in (Starzyk and Horzyk, 2019a). As shown in Figure 6, the Levenshtein distance of all models increases with an increasing number of sentences. However, our model always outperforms the other two methods. For example, when the number of training sentences is 500, the Levenshtein distances of LSTM and ANAKG are both above 2.5, while the Levenshtein distance of the proposed spiking model is below this threshold. In addition, the results also show that with more neurons in each column, a better retrieval performance can be obtained.

**Figure 7:**
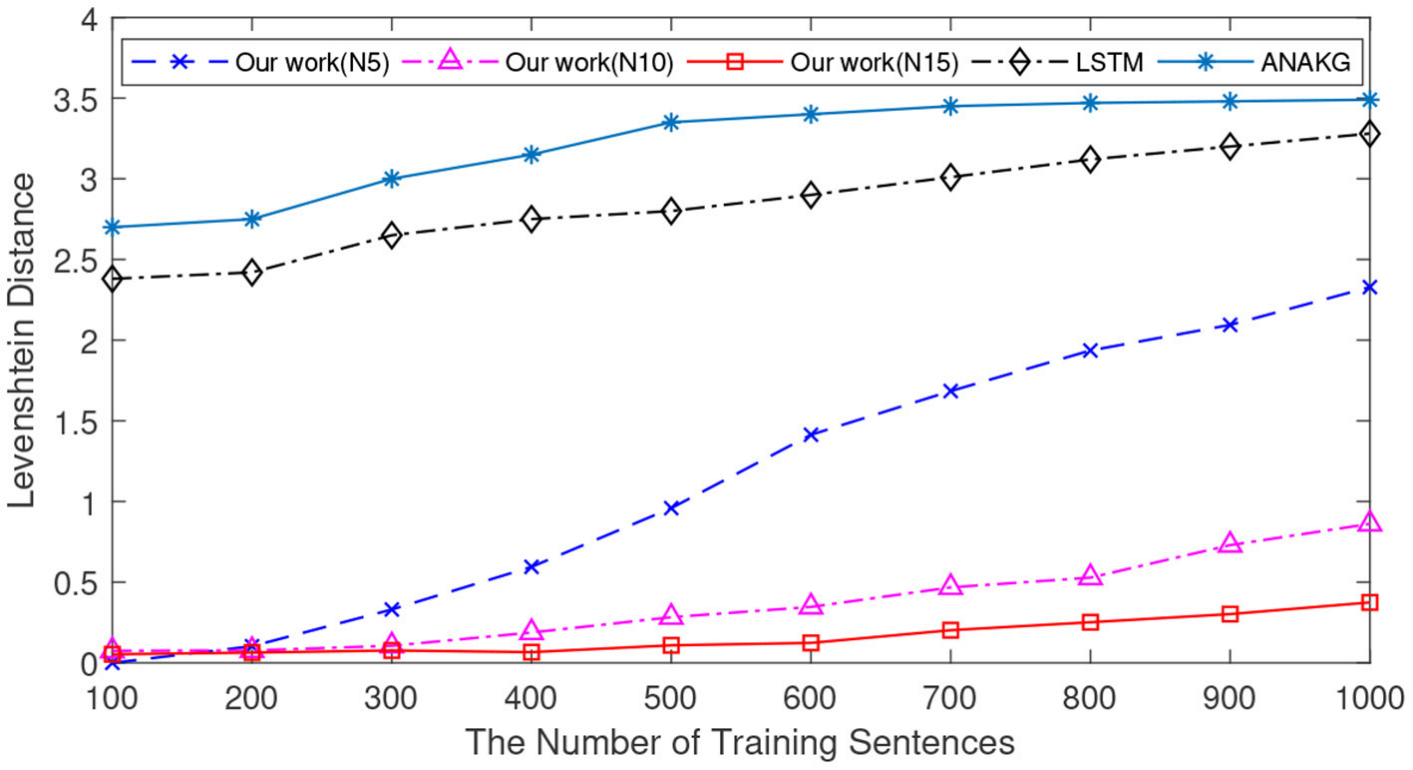
The memory retrieval performance of the LSTM, the ANAKG, and our proposed model with different number of unique words. Our model is trained with different column size and “N5” means there are 5 neurons in each column.

Since different sentences may consist of the same words, the number of unique words is different from that of the sentence. The number of unique words is also an important index to verify the memory capacity (Starzyk and Horzyk, 2019a). Next, we conduct experiments to verify the Levenshtein distance as a function of the number of unique words, and the experimental settings are the same as in previous experiments. In this experiment, the number of unique words varies from 100 to 1,000 with an interval of 100. Figure 7 shows the mean Levenshtein distances of different methods. It exhibits a similar pattern to the results in Figure 6. First of all, when the number of unique words increases, it is more difficult to retrieve the memorized sentences for all memory models. Secondly, the proposed method still outperforms the other two methods.

#### 3.4.2. Effect of the Length of Sentences

**Figure 8:**
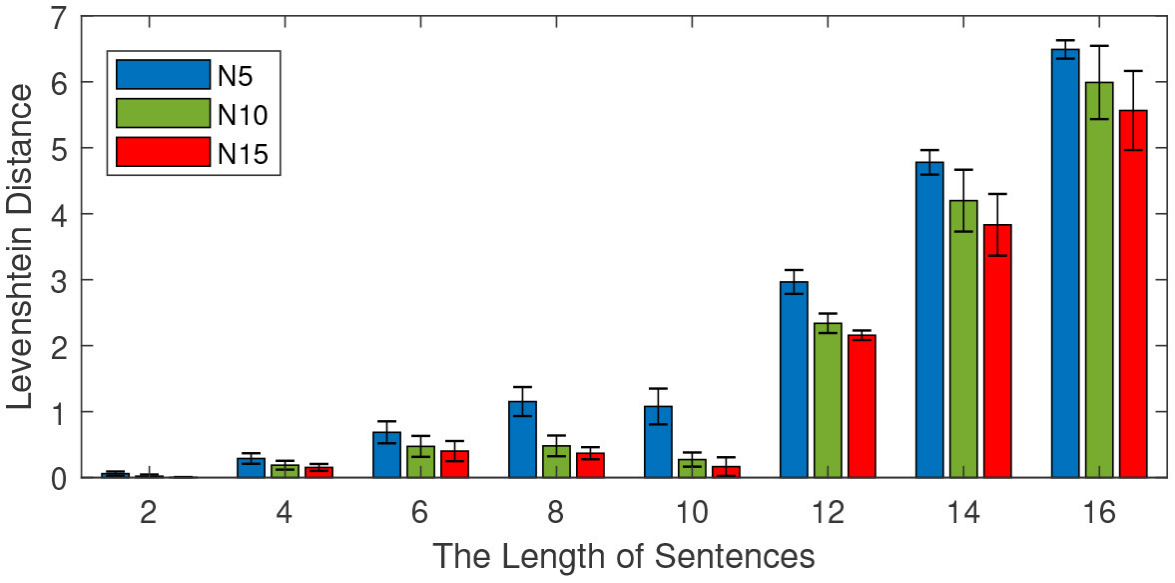
Effect of the length of sentences on retrieval performance. The total number of sentences is 100, the number of neurons in each column is 5, 10, and 15. The length of sentences varies from 2 to 16 with an interval of 2, and the length of the input is half of the training length.

This experiment is conducted to investigate the effect of the sentence length on memory capability. We train the model with different lengths of learning sentences from the CBT dataset. The number of training sentences is 100, and the length of sentences varies from 2 to 16 with an interval of 2. Different mini-column sizes (5 neurons, 10 neurons, and 15 neurons) are investigated and reported. For each length, 10 independent experiments are conducted to obtain the average performance. After learning, half of the learning sentences are presented to the model to see the retrieval performance. The experimental results are shown in Figure 8, in which both the average performance (mean values) and the standard deviations are reported.

As shown in Figure 8, the Levenshtein distance increases with the increase of the lengths of training sentences. For example, when the length of training sentences is 10, all models achieve a Levenshtein distance below 2. However, if the length of the sentence is 16, the Levenshtein distances of all methods are above 5. This means a longer length of sentence is more difficult for all methods. On the other hand, it is noteworthy to find that the more neurons there are in each column, the better the retrial performance becomes. This observation is very useful for the design of the memory model.

#### 3.4.3. Effect of the Input Length

**Figure 9:**
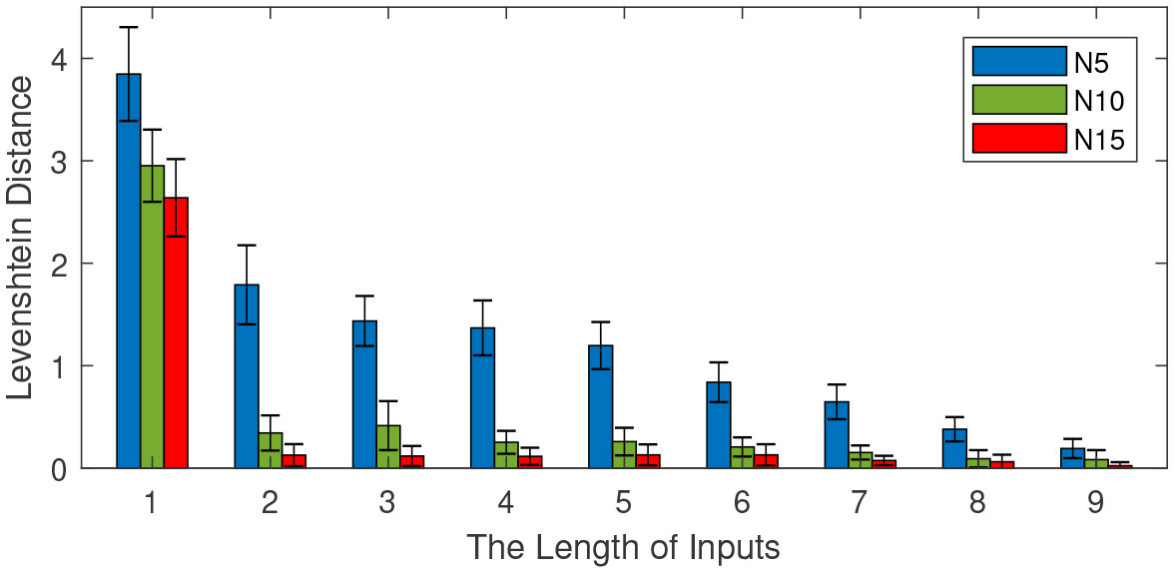
Effect of the input length on memory retrieval performance. The number of total sentences is 100 and the number of neurons in each column is 5, 10, and 15. The length of inputs varies from 1 to 9 with an interval of 1.

In these experiments, we investigate the effect of the input length on retrieval performance. We first train the memory model with the first eleven words of sentences. Then, we test the retrieval capability by presenting different lengths of inputs. The length of inputs varies from 1 to 9 with an interval of 1. The other parameters are set as follows: the number of total sentences is 100 and the number of neurons in each column various from 5 to 15 with an interval of 5. For each input length, 10 independent experiments are conducted to obtain the average performance. The retrieval performance is shown in Figure 9.

Figure 9 shows the effect of the input length on memory retrieval performance. The Levenshtein distance decreases with the increase of input length. This means a longer input content contributes to a better memory retrieval. When the length of the input is 1, the Levenshtein distances of models with different sizes are all above 2. However, with an input length of 9, the Levenshtein distances are all below 0.5. On the other hand, the size of the mini-column still plays a very important role in memory retrieval. A bigger size mini-column results in a better performance.

## 4. Discussion and Conclusion

The formation of memory is a brain-wide complex process, and it is extremely important in various cognitive tasks. Although the exact mechanism of the formation of memory remains unclear, researchers have devoted significant effort to proposed different models to simulate the human memory system. Among these methods, the SNN-based models are more attractive due to the biological plausibility and energy-efficiency. This work is one of the SNN-based memory models. Experimental results demonstrate that the proposed model can effectively store a huge number of data and can retrieve them with higher accuracy as compared with the existing memory models. This work not only provides a new memory model but also provides suggestions of how the brain formulates memory with various biological mechanisms, such as excitatory/inhibitory neurons, STDP, spike-based encoding, and mini-column structure.

In terms of biological plausibility, the structure of the proposed memory model has inspired the human neocortex that is formed by many mini-column. This structure is very important to improve memory capacity and distinguish different contexts. In addition, the biologically plausible spiking neuron is used to construct the memory model, it can better simulate the dynamics of a real neuron. Finally, the model considers the neuronal diversity in the neural system. Both the excitatory and inhibitory neurons are employed in the memory model to improve memory performance. As we demonstrated in 2, the inhibitory neuron plays a very important role in preventing the wrong prediction and contributes to a better performance in retrieval. In terms of the energy-efficiency, this work applies the temporal-coding mechanism whereby information is represented by a single spike from the neuron. Compared to spike rate-based methods that use the number of spikes to represent information, our method greatly reduces the energy requirement. In addition, the model can be trained in a one-shot learning manner. Therefore, the learning efficiency is much higher than other methods that require hundreds of iterations.

Our model can still be improved from the following aspects. First of all, a sparsely distributed encoding scheme can be employed to replace the existing one-hot encoding scheme. With the sparsely distributed coding scheme, the memory capacity can be further improved. Secondly, we can build a hierarchical model to perform more complex memory and cognitive tasks, such as remembering a song. Thirdly, it will be very interesting to implement the proposed learning strategy and model to neuromorphic hardware platforms, such as Loihi (Davies et al., 2018) and Tianjic (Pei et al., 2019).

## Data Availability Statement

The raw data supporting the conclusions of this article will be made available by the authors, without undue reservation.

## Author Contributions

YL and XW proposed the main idea. All authors conducted the experiments and wrote the manuscript.

## Conflict of Interest

The authors declare that the research was conducted in the absence of any commercial or financial relationships that could be construed as a potential conflict of interest.
